# Association of the standardized phase angle with the incidence and severity of acute kidney injury after cardiac surgery

**DOI:** 10.1097/MD.0000000000044239

**Published:** 2025-08-29

**Authors:** Mauricio Carvallo-Venegas, Jorge Andrade-Sierra, Enrique Rojas-Campos, Rolando Claure-Del Granado, Miguel Medina-Pérez, Luis Gerardo González-Correa, José Ignacio Cerrillos-Gutiérrez, Adriana Banda-López, Ricardo Parra-Guerra, Alfredo Gutiérrez-Govea, Saúl Tejeda-del Toro, Laura Elizabeth Aguilar-Fletes, Francisco Gerardo Yanowsky-Escatell, Moises Cruz-Landino, Luis Alberto Evangelista-Carrillo

**Affiliations:** a Department of Nephrology and Organ Transplant Unit, Specialties Hospital, National Western Medical Centre, Mexican Social Security Institute, Guadalajara, Jalisco, Mexico; b Department of Physiology, University Health Sciences Center, University of Guadalajara, Guadalajara, Jalisco, Mexico; c Medical Research Unit in Renal Diseases, Specialties Hospital, National Western Medical Centre, Mexican Social Security Institute, Guadalajara, Jalisco, Mexico; d Division of Nephrology, Hospital Obrero No-2 Caja Nacional de Salud, Cochabamba, Bolivia; e Department of Health Sciences-Illnes as an Individual Process, University Center of Tonala, University of Guadalajara, Guadalajara, Jalisco, Mexico.

**Keywords:** acute kidney injury, bioelectrical impedance, cardiac surgery, standardized phase angle

## Abstract

Bioelectrical impedance permits the measurement of variables related to morbidity and mortality in cardiac surgery patients. The preoperative phase angle (PhA) is one of the variables associated with increased mortality, prolonged duration of invasive mechanical ventilation, and prolonged hospital stay. However, its relationship with acute kidney injury (AKI) is unknown. A prospective cohort study measured the PhA 24 hours before surgery, and the standardized phase angle (SPhA) was calculated from data from the reference population. The incidence and severity of AKI were determined over 7 days after surgery. Multivariate analysis was performed to determine the relationships between PhA or SPhA and the incidence and severity of AKI after cardiac surgery. A total of 120 patients were recruited. The incidence of AKI during the 7 days after cardiac surgery was 37%, 70% of which were classified as Kidney Disease Improving Global Outcomes (KDIGO) 1, 19% as KDIGO 2, and 11% as KDIGO 3. SPhA > −0.54 was associated with a lower incidence of AKI at 21% and lower AKI KDIGO 1 at 8% (2/24 patients) than SPhA ≤ −0.54 was associated with an AKI incidence of 40% (*P* = .072) and AKI KDIGO 1 incidence of 30% (29/96 patients) (*P* = .029). There were no differences in the incidence of AKI classified as KDIGO 2–3. According to the multivariate analysis, an SPhA > −0.54 was a protective factor (RR 0.254, CI 0.074–0.870, *P* = .029). A high SPhA (>−0.54) serves as a protective factor against AKI, particularly against AKI classified as KDIGO 1.

## 1. Introduction

Acute kidney injury (AKI) is a significant and common complication following cardiac surgery,^[1]^ with incidence rates varying between 7.9%^[2]^ and 43%,^[3,4]^ depending on the definition used, the population studied, and the study design. In addition to its frequency, AKI is associated with a significant increase in mortality in the postoperative period following cardiac surgery.^[2,4,5]^ In a multicentric study of 3460 patients, the occurrence of AKI after cardiac surgery was independently associated with a 4-fold increase in mortality during hospitalization.^[6]^ Given the importance of postoperative AKI in medical practice, identifying its risk factors is crucial for effective monitoring and prevention.

There are a wide number of risk factors associated with AKI after cardiac surgery.^[7]^ The most important risk factors are previous chronic kidney disease (CKD), postoperative hypotension, >140 minutes spent on cardiopulmonary bypass, advanced age, diabetes, and previous congestive heart failure.^[8]^ Various predictive models of AKI have been designed using different combinations of these risk factors, achieving moderate success.^[9,10]^ These tools have the potential to aid in decision-making, enhance patient care, and facilitate the investigation of preventative or therapeutic strategies.^[9]^ Another tool that could be used to predict mortality in postoperative cardiac surgery patients is bioelectrical impedance, or bioimpedance (BI). It has several advantages, including the ability to be utilized at the bedside. It is portable, simple, noninvasive, reproducible, and fast to use.^[11]^

BI is a technique that permits the electrical properties of tissues to be used to estimate their composition and obtain data at the cellular level.^[11]^ By using the BI to determine the reactance and resistance of an organism, inferences can be made about the organism’s hydration, nutritional, and pathological status.^[11]^ A variable that is obtained from the relationship between reactance and resistance is the phase angle (PhA), which, in recent decades, has demonstrated its value in clinical practice.^[12]^ The PhA represents the reduction in current as it passes through the cellular membrane, or, in other words, the phase shift of the electrical current waveform as it changes transmission media.^[11,12]^ A high PhA indicates a greater total cellular mass and better integrity of the cell membrane.^[11,12]^ In various studies, PhA has been linked to morbimortality variables and validated as a prognostic factor for morbimortality in different groups.^[13]^

In a prospective cohort of 50 elective cardiac surgery patients in Brazil, preoperative PhA was inversely correlated with less postoperative mechanical ventilation time and length of hospital stay.^[14]^ In another cohort of 642 patients, the preoperative PA was an independent predictive variable for the need for blood transfusion during the postoperative cardiac surgery period.^[15]^ Additionally, in a cohort of 227 major cardiac surgery patients, a lower PhA before surgery was associated with a higher risk of mortality at 1 month and 12 months and a longer hospital stay.^[16]^ In that same study, patients with a PhA ≤ 4.5° had a greater incidence of AKI than did those with a PhA of 4.6° to 5.5° or ≥5.6°, but the difference was not statistically significant.^[16]^ The objective of this study was to determine the associations of the preoperative PhA and standardized phase angle (SPhA), obtained via the BI, with the incidence and severity of AKI in the 7 days following cardiac surgery. The hypothesis was that patients with a greater preoperative PhA or SPhA would have a lower incidence and severity of AKI after cardiac surgery.

## 2. Patients and methods

Between August 2022 and October 2023, a prospective cohort study enrolled 120 adult patients from the Department of Cardiac Surgery at the Specialties Hospital of the National Western Medical Center of the Mexican Institute of Social Security in Guadalajara, Jalisco, Mexico. Each patient underwent cardiac surgery (valve replacement, coronary bypass, or both) during a single elective operation. Patients with the following conditions were excluded: cardiac implant devices, metallic valves, prior AKI, kidney transplant, CKD with a glomerular filtration rate (GFR) ≤ 30 mL/min/1.73 m², anasarca, amputation of any extremity, or severe neuropsychiatric disturbances affecting cooperation with study procedures.

### 2.1. Procedures

The observation period commenced with the measurement of PhA within 24 hours before cardiac surgery and ended 7 days postsurgery. PhA measurements were conducted via the Body Composition Monitor edition 9/01.13 with software version 3.3× (Fresenius Medical Care AG & Co. KGaA, Bad Homburg, Germany). This device applies the BI technique via spectroscopy and is grounded in 2 validated physiological models: the volume model^[17]^ and the body composition model.^[18]^ The PhA measurement was conducted by a trained researcher at BI, following the manufacturer’s instructions. This included ensuring that the patient fasted for 4 hours, avoiding simultaneous use of other electrical contact devices such as electrocardiograms, positioning the patient supine on a flat, nonconductive surface, and ensuring that the patient did not have a temperature ≥38 °C. Measurements were taken at a frequency of 50 kHz.

The SPhA, which allows adjustments for age, sex, and body mass index (BMI), was calculated via the following formula: ([phase angle measurement − average phase angle of the reference population]/standard deviation of the reference population). The reference population was the Mexican population according to the Espinosa^[19]^ database.

The following data from the physical and electronic files were collected: patient age, sex, congestive heart failure (CHF), left ventricular ejection fraction (LVEF) < 35%, preoperative intra-aortic balloon pump, chronic obstructive pulmonary disease (COPD), history of diabetes or hypertension, use of medications (insulin), previous cardiac surgery, type of cardiac surgery, preoperative GFR, Cleveland score, use of a cardiopulmonary bypass pump, length of time on cardiopulmonary bypass, length of time of aortic compression, serum albumin, and grip strength. Preoperative and postoperative serum creatinine (SCr) levels were assessed via the colorimetric method in the laboratory. The preoperative GFR was calculated via the CKD-EPI formula. The Cleveland score was calculated on the basis of the original study.^[20]^ The Cleveland score is a model that has been validated for the prediction of AKI after cardiac surgery.^[9,20]^ After the PhA was obtained, it was classified as having a high value of >4.5° or a low value of ≤4.5°. The cutoff point was selected using the study by Mullie et al,^[16]^ in which the lowest tertile that had a PhA ≤ 4.5 had a higher incidence of AKI. A cutoff point was also selected for SPhA, which was determined by classifying the population into quintiles. The cutoff was based on our data, since we could not find one in the previous literature. Following surgical intervention, the measurement of SCr was performed on the basis of the protocol of the unit and was measured at a frequency of at least once every 24 hours during the patient’s postoperative stay in the intensive care unit. The highest postoperative SCr was recorded in the first 48 hours and over the first 7 days after surgery. The use of renal replacement therapy (RRT) and mortality were recorded 7 days after surgery by consulting the patients’ electronic charts. The data obtained were stored in a secure database for analysis (File S1, Supplemental Digital Content, https://links.lww.com/MD/P828).

### 2.2. Definitions

We considered the following definitions of AKI based on the Kidney Disease Improving Global Outcomes (KDIGO) Guidelines^[21]^: an increase in SCr of 0.3 mg/dL from baseline in a period of 48 hours and/or an increase of ≥1.5 times the baseline value in a period of 7 days after cardiac surgery; the patient’s baseline creatinine was considered the most recent SCr obtained in the 3 months prior to surgery. The urinary output criterion was not considered because of its variability in measurement among individuals. AKI was classified according to the guidelines set out by KDIGO.^[21]^

### 2.3. Sample size

The sample size was calculated via the formula for proportions with a 95% confidence level. An expected AKI incidence of 12% and a difference in incidence between groups of 18% were assumed on the basis of data from Mullie et al.^[16]^ The initial calculation yielded 50 subjects per group, with an additional 20% added for potential losses, resulting in a final N value of 60 per group and a total of 120 subjects.

### 2.4. Statistical analysis

The data are presented as the means ± standard deviations or medians with interquartile ranges and as numbers and percentages where appropriate. The normality of the distribution of the variables was evaluated with the Kolmogorov–Smirnov test. To compare categorical variables, the chi-squared test was used. In the case of expected values <5, Fisher exact test was used. To compare quantitative variables, Student *t* test or the Mann–Whitney *U* test was used, depending on the distribution. The Pearson correlation coefficient was used in the analysis to determine the relationship between PhA before cardiac surgery and AKI after cardiac surgery. Spearman rank correlation coefficient was used to determine the relationship between SPhA before cardiac surgery and AKI after cardiac surgery. Logistic regression analysis was performed using parameters with a *P* value of ≤.05 in the univariate analysis and variables related in previous studies to AKI to determine the predictive factors for AKI. SPSS version 26 software (IBM Corp., Armonk ) was used for the statistical analysis. A value of *P* ≤ .05 was considered statistically significant.

### 2.5. Ethical considerations

The study adheres to the ethical principles for research in humans established by the World Declaration of Helsinki and the regulations outlined by the General Health Law for Research in Mexico. Before recruitment, potential study participants were provided clear and comprehensive information about the study, and written informed consent was obtained from all of the participants. The study protocol was reviewed and approved by the local Ethics and Research Committee of the Health Research Center of the Specialties Hospital, National Western Medical Centre, Mexican Social Security Institute, Guadalajara, Jalisco, Mexico, under registration number *R*-2023-1301-076.

## 3. Results

A total of 120 patients were recruited, with an average age of 65 years (interquartile range [IQR], 57–69), and these patients were predominantly male (63%). Demographic characteristics, comorbidities, surgical details, laboratory tests, anthropometry, and PhA are summarized in Table 1. The most common comorbidities were diabetes (43%) and CHF (18%). Coronary bypass surgery (49%) and valvular surgery (42%) were the most common procedures, with both being performed in 9% of the patients during a single surgical operation. Prior to surgery, 87% of patients had a GFR ≥ 60 mL/min/1.73 m². Using a Cleveland score cutoff of ≥3, as recommended by Kiers et al,^[9]^ 26% of patients were deemed at higher risk for AKI.

**Table 1:** Demographic characteristics, surgical information, laboratory tests, anthropometry, and phase angle.

	Frequency, average, or median N = 120	Standard deviation or interquartile range
Age (yr)	65	IQR, 57–69
Masculine sex n (%)	76 (63)	
CHF n (%)	22 (18)	
LVEF < 35% n (%)	8 (7)	
COPD n (%)	1 (1)	
Diabetes n (%)	52 (43)	
Diabetes with insulin n (%)	28 (23)	
Previous cardiac surgery n (%)	3 (3)	
Intra-aortic balloon pump	0 (0)	
Type of surgery		
Bypass n (%)	59 (49)	
Valvular n (%)	50 (42)	
Both n (%)	11 (9)	
Cardiopulmonary bypass (%)	119 (99)	
Time on cardiopulmonary bypass (min)	114	IQR, 93–153
Aortic compression (min)	91	IQR, 74–118
Serum albumin (g/dL)	4	IQR, 3.7–4.3
Serum creatinine (mg/dL)	0.87	IQR, 0.7–1.0
GFR (mL/min/1.73 m^2^)	91	IQR, 74–101
Cleveland score		
0–2 n (%)	89 (74)	
3–17 n (%)	31 (26)	
Weight (kg)	72	±14
BMI (kg/m^2^)	26	IQR, 24–29
MAP (mm Hg)	82	±10
Grip strength (kg)	24.80	±8.23
Phase angle		
≤4.5° n (%)	17 (14)	
>4.5° n (%)	103 (86)	
Phase angle (°)	5.6	±1
Standardized phase angle	−1.35	IQR, −2.3 to −0.68
AKI at 7 days n (%)	44 (37)	
AKI KDIGO 1 n (%)	31 (26)	
AKI KDIGO 2 n (%)	8 (7)	
AKI KDIGO 3 n (%)	5 (4)	
AKI and received RRT n (%)	1 (1)	
Mortality at 7 days n (%)	16 (13)	

AKI = acute kidney injury, BMI = body mass index, CHF = congestive heart failure, COPD = chronic obstructive pulmonary disease, GFR = glomerular filtration rate, KDIGO = Kidney Disease Improving Global Outcomes, LVEF = left ventricular ejection fraction, MAP = mean arterial pressure, RRT = renal replacement therapy.

The incidence of AKI within 7 days postsurgery was 37%, categorized as KDIGO 1 in 70%, KDIGO 2 in 19%, and KDIGO 3 in 11%, with only 1% requiring RRT. The mortality rate within 7 days postsurgery was 13%.

### 3.1. Phase angle

When the data were stratified by the PhA value, 14% had a PhA ≤ 4.5°. Compared with patients with PhA > 4.5° (Table 2), those with PhA ≤ 4.5° were older (70 years, IQR 63–75 vs 63 years, IQR 57–68, *P* = .014), less frequently male (41% vs 67%, *P* = .041), weighed less (60 kg ± 10 vs 74 kg ± 14, *P* = .000), had a lower BMI (23.3 kg/m², IQR 22.7–24.4 vs 27.1 kg/m², IQR 24.6–29.0, *P* = .000), and had weaker grip strength (18 kg ± 7 vs 26 kg ± 9, *P* = .001). There was also a trend toward a greater incidence of CHF (35% vs 16%, *P* = .051) and lower serum ALB levels (3.8 g/dL, IQR 3.4–4.1 vs 4.1 g/dL, IQR 3.8–4.3, *P* = .065) in the ≤4.5° group. Although AKI incidence (47% vs 35%, *P* = .377) and mortality (24% vs 12%, *P* = .182) were greater in the ≤4.5° group, these differences were not statistically significant.

**Table 2:** Comparison of characteristics and outcomes between the groups with high and low PhA.

	PhA ≤ 4.5° N = 17	PhA > 4.5° N = 103	*P* value
Age (yr)	70 (IQR, 63–75)	63 (IQR, 57–68)	.014
Masculine sex n (%)	7 (41)	69 (67)	.041
CHF n (%)	6 (35)	16 (16)	.051
LVEF < 35% n (%)	1 (6)	7 (7)	1.000
COPD n (%)	0 (0)	1 (1)	1.000
Diabetes n (%)	9 (53)	43 (42)	.388
Diabetes with insulin n (%)	5 (29)	23 (22)	.522
Previous cardiac surgery n (%)	0 (0)	3 (3)	1.000
Type of surgery			.291
Bypass n (%)	6 (35)	53 (51)	
Valvular n (%)	8 (47)	42 (41)	
Both n (%)	3 (18)	8 (8)	
Time on cardiopulmonary bypass, minutes	105 (IQR, 91–185)	115 (IQR, 95–149)	.637
Aortic compression, minutes	91 (IQR, 73–151)	91 (IQR, 75–115)	.512
Serum albumin (g/dL)	3.8 (IQR, 3.4–4.1)	4.1 (IQR, 3.8–4.3)	.065
Serum creatinine (mg/dL)	0.8 (IQR, 0.7–1.0)	0.9 (IQR, 0.7–1.0)	.390
TFG (mL/min/1.73 m^2^)	88 (IQR, 68–96)	91 (IQR, 74–101)	.397
Cleveland score ≥ 3 n (%)	7 (41)	24 (23)	.119
Weight (kg)	60 ± 10	74 ± 14	.000
BMI (kg/m^2^)	23.3 (IQR, 22.7–24.4)	27.1 (IQR, 24.6–29)	.000
MAP (mm Hg)	78 ± 10	82 ± 9	.092
Grip strength (kg)	18 ± 7	26 ± 9	.001
AKI at 7 days n (%)	8 (47)	36 (35)	.377
AKI KDIGO 1 n (%)	5 (29)	26 (25)	.716
AKI KDIGO 2 and 3 (%)	3 (18)	10 (10)	.394
AKI that required RRT n (%)	0 (0)	1 (1)	1.000
Mortality at 7 days n (%)	4 (24)	12 (12)	.241

AKI = acute kidney injury, BMI = body mass index, CHF = congestive heart failure, COPD = chronic obstructive pulmonary disease, GRF = glomerular filtration rate, KDIGO = Kidney Disease Improving Global Outcomes, LVEF = left ventricular ejection fraction, MAP = mean arterial pressure, PhA = phase angle, RRT = renal replacement therapy.

### 3.2. Standardized phase angle

**Figure 1.**
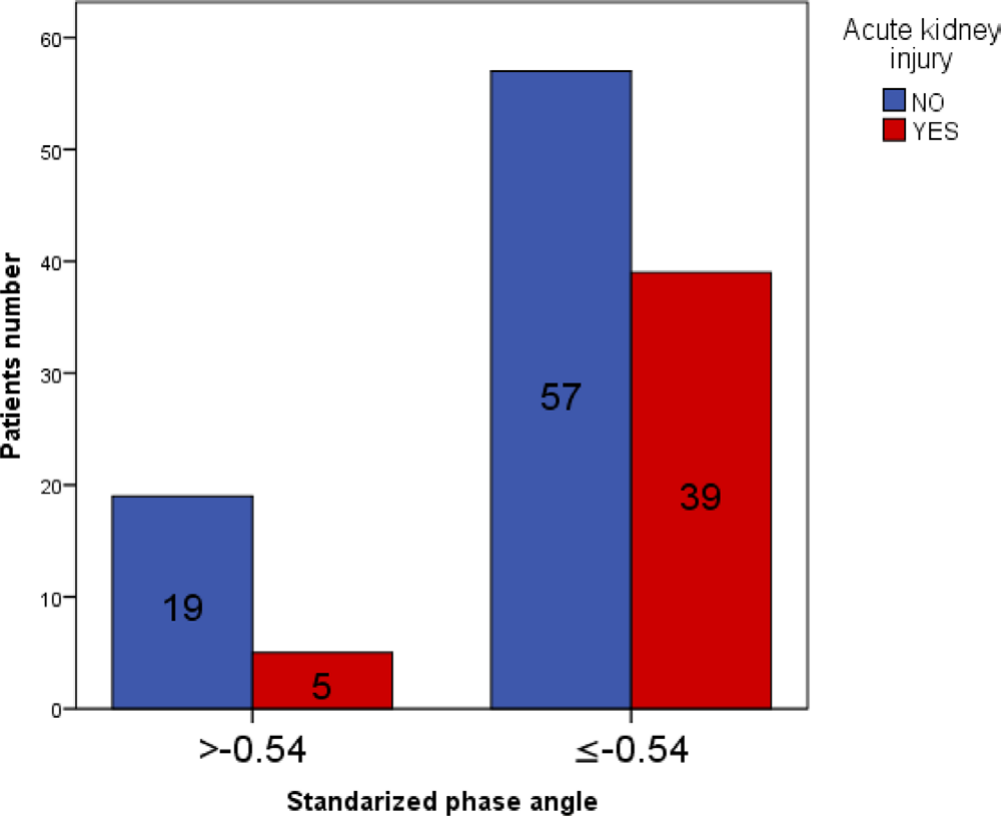
Comparison of the incidence of AKI in patients with high and low SPhA. The incidence of AKI was lower in the SPhA ≥ −0.54 group (21%) than in the SPhA < −0.54 (40%) group, although the difference was not statistically significant (*P* = .072). AKI = acute kidney injury, SPhA = standardized phase angle.

**Figure 2.**
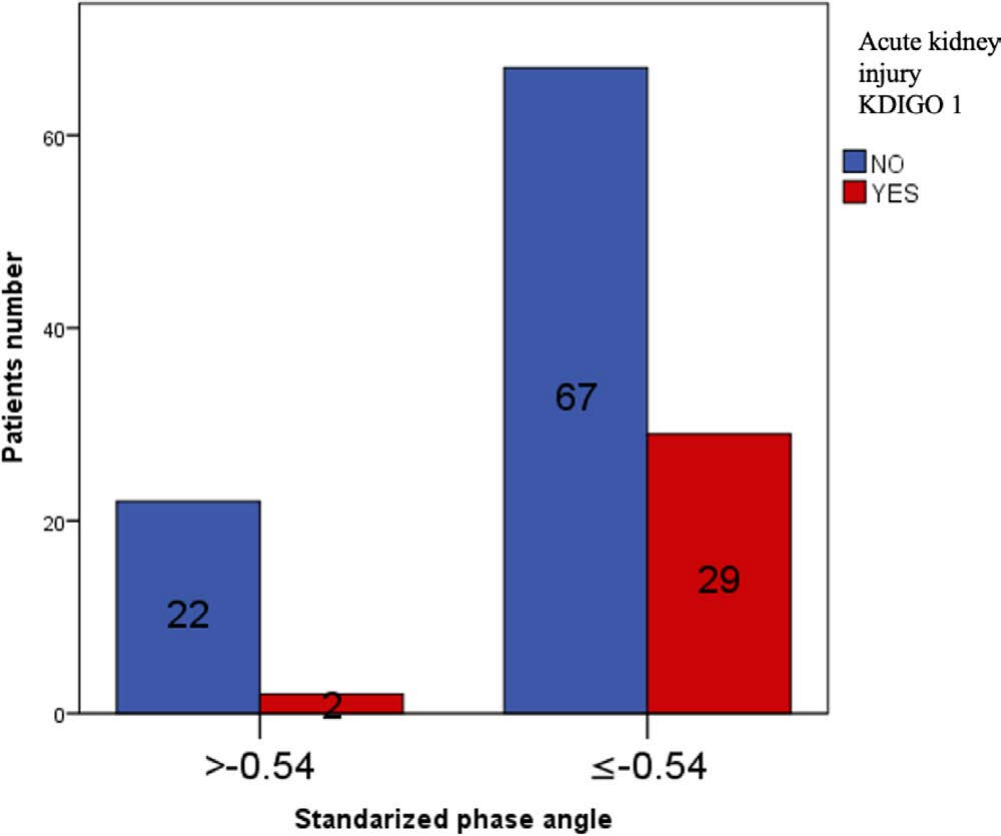
Comparison of the incidence of AKI KDIGO 1 in patients with high and low SPhA. The incidence of AKI KDIGO 1 was lower in the SPhA ≥ −0.54 group (8%) than in the SPhA < −0.54 group (30%) (*P* = .029). AKI = acute kidney injury, KDIGO = Kidney Disease Improving Global Outcomes, SPhA = standardized phase angle.

A comparison of the groups in which SPhA was used with a cutoff of −0.54 (Table 3) revealed that those with SPhA ≥ −0.54 had higher serum albumin levels (4.2 g/dL, IQR 4.1–4.5 vs 4.0 g/dL, IQR 3.7–4.2, *P* = .028). There were no significant differences in demographic characteristics, comorbidities, surgical details, or other laboratory or anthropometry measures between the groups. The incidence of AKI was lower in the SPhA ≥ -0.54 group (21%) than in the SPhA < −0.54 (40%) group, although the difference was not statistically significant (*P* = .072, Fig. 1). Specifically, the incidence of KDIGO 1 AKI was significantly lower in the SPhA ≥ −0.54 group (8%) than in the SPhA < −0.54 group (30%), *P* = .029 (Fig. 2). There were no significant differences in the incidence of KDIGO 2–3 AKI (13% vs 10%, *P* = .722) or the need for RRT (0% vs 1%, *P* = 1.000) between these groups. The odds ratio for KDIGO 1 AKI between SPhA ≥ −0.54 vs SPhA < −0.54 was 0.21 (95% CI 0.05–0.95), and the relative risk was 0.26 (95% CI 0.06–1.05). Mortality within 7 days postsurgery did not significantly differ between the SPhA ≥ −0.54 group and the SPhA < −0.54 group (17% vs 13%, *P* = .737).

**Table 3:** Comparison of characteristics and outcomes between the groups with high and low SPhA.

	SPhA ≥ −0.54 N = 24	SPhA < −0.54 N = 96	*P* value
Age (yr)	61 (IQR, 55–66)	65 (IQR, 59–69)	.136
Masculine sex n (%)	13 (54)	63 (66)	.279
Congestive heart failure n (%)	3 (13)	19 (20)	.560
LVEF < 35% n (%)	1 (4)	7 (7)	1.000
COPD n (%)	0 (0)	1 (1)	1.000
Diabetes n (%)	9 (38)	43 (45)	.519
Diabetes with insulin n (%)	4 (17)	24 (25)	.590
Previous cardiac surgery n (%)	2 (8)	1 (1)	.102
Type of surgery			.139
Bypass n (%)	11 (46)	48 (50)	
Valvular n (%)	13 (54)	37 (39)	
Both n (%)	0 (0)	11 (11)	
Time on cardiopulmonary bypass (min)	105 (IQR, 94–137)	120 (IQR, 94–157)	.234
Aortic compression (min)	89 (IQR, 74–103)	92 (IQR, 74–123)	.335
Serum albumin (g/dL)	4.2 (IQR, 4.1–4.5)	4.0 (IQR, 3.7–4.2)	.028
Serum creatinine (mg/dL)	0.9 (IQR, 0.7–1.1)	0.9 (IQR, 0.7–1.0)	.798
GFR (mL/min/1.73 m^2^)	91 (IQR, 73–101)	91 (IQR, 74–100)	.839
Cleveland score ≥ 3 n (%)	5 (21)	26 (27)	.532
Weight (kg)	72 ± 10	72 ± 15	.774
BMI (mL/min/1.73 m^2^)	27 (IQR, 25–30)	26 (IQR, 24–28)	.213
MAP (mm Hg)	81 ± 9	82 ± 10	.479
Grip strength (kg)	26 ± 10	25 ± 9	.710
AKI at 7 days n (%)	5 (21)	39 (40)	.072
AKI KDIGO 1 n (%)	2 (8)	29 (30)	.029
AKI KDIGO 2-3 n (%)	3 (13)	10 (10)	.722
AKI that required RRT n (%)	0 (0)	1 (1)	1.000
Mortality at 7 days n (%)	4 (17)	12 (13)	.737

AKI = acute kidney injury, BMI = body mass index, CHF = congestive heart failure, COPD = chronic obstructive pulmonary disease, GFR = glomerular filtration rate, KDIGO = Kidney Disease Improving Global Outcomes, LVEF = left ventricular ejection fraction, MAP = mean arterial pressure, RRT = renal replacement therapy.

### 3.3. Correlations and multivariate analysis

Pearson correlation analysis revealed no significant association between PhA and AKI (*P* = .357). Spearman analysis revealed a negative correlation (*R* = −0.164, *P* = .073) between SPhA as a dichotomous variable (cutoff ≥ −0.54) and AKI. Univariate analysis revealed that older age, lower GFR, Cleveland score ≥ 3, and higher weight were significantly associated with increased AKI frequency (all *P* < .05). In the multivariate analysis, the factors along with diabetes, LVEF < 35%, time on cardiopulmonary bypass, aortic compression time, and SPhA ≥ −0.54 were included. The model (Table 4) identified the GFR as a protective factor, with a nearly 4% decrease in AKI risk for every 1 mL/min/1.73 m² increase (95% CI 2–6%, *P* = .001). An SPhA > −0.54 was also protective, reducing AKI risk by 74% (95% CI 13–92%, *P* = .029). Weight tended to increase AKI risk (3% per additional kg, 95% CI 0–7%, *P* = .062). Patients with AKI had significantly greater postoperative mortality (25% vs 7%, *P* = .004) than did those without AKI, and mortality was highest in the group with KDIGO 2–3 AKI (46% vs 9%, *P* = .004).

**Table 4:** Multivariate analysis for the prediction of AKI.

Multivariate analysis			
*X*^2^ = 22.995; *P* < .0001			
Variable	Risk	CI 95%	*P*-value
GFR (mL/min/1.73 m^2^)	0.961	0.939–0.984	.001
SPhA > −0.54	0.254	0.074–0.870	.029
Weight (kg)	1.032	0.998–1.066	.062

Covariables: age and length of time on cardiopulmonary bypass.

AKI = acute kidney injury, GFR = glomerular filtration rate, SPhA = standardized phase angle.

## 4. Discussion

The incidence of AKI in the present study cohort was 37%, and the need for RRT was <5%, which is consistent with other studies.^[2–4]^ The most common stage of AKI was stage 1, which occurred in 70% of the patients; this pattern is consistent with findings from 19 other studies.^[22]^ The demographic characteristics of the study population, with an average age of 65 years and a predominance of males, were typical for a population requiring cardiac surgery.^[16,23,24]^ Additionally, the incidence of comorbidities, such as diabetes and CHF, was similar to that reported by Mullie et al.^[16]^ The incidence of CKD was lower than that reported in the aforementioned study (13% vs 28%), which aligns with the rates reported in the general population.^[25]^ This difference is attributed to the exclusion of patients with a GFR < 30 mL/min/1.73 m² from the present study.

We demonstrated an association between PhA and patient age, sex, weight, and grip strength. This association has been described in other studies in patients prior to cardiac surgery; for example, in the study by Ryz et al^[26]^ patients with PhA ≤ 5.84° had a greater prevalence of female sex and greater age. In the study by Visser et al^[27]^ a PhA ≤ 5.38° was associated with a lower BMI and lower grip strength. In this study, a PhA cutoff of ≤4.5° was consistently associated with various variables. Although there was a greater incidence of mortality at 7 days in the low PhA group than in the high PhA group (24% vs 12%, respectively), this difference was not statistically significant (*P* = .241). While Mullie et al^[16]^ reported a significant association between PhA and mortality at 1 and 12 months postsurgery, our study focused on mortality within 7 days postsurgery and did not find a statistically significant difference between groups (24% vs 12%, respectively; *P* = .241). Notably, our study may not have had sufficient statistical power or follow-up duration to accurately assess the association between preoperative PhA and postoperative mortality.

In Mullie et al,^[16]^ there was a greater incidence of AKI in the group with a PhA ≤ 4.5° than in our study, but the difference (47% vs 35%, respectively) was not statistically significant (*P* = .377). This discrepancy could be attributed to different AKI definitions; our study adhered to the KDIGO Guidelines^[21]^ (excluding urinary output criteria) and focused on a 7-day postoperative diagnostic window, which may provide a more precise delineation of AKI associated with cardiac surgery. In contrast, Mullie et al^[16]^ did not specify the AKI definition used and assessed AKI over the entire hospital stay, potentially overestimating the relationship between preoperative PhA and AKI.

Although PhA did not demonstrate an association with AKI risk in our study, SPhA (adjusted for age, sex, and BMI) was significantly associated with AKI risk, particularly for stage 1 AKI (being protective when SPhA > −0.54). Several hypotheses could explain this association. First, the PhA may be correlated with hemodynamic stability during cardiac surgery. In a single-center cohort of 168 patients, those with lower average PhA values tended to have greater fluid balance on the day of surgery than those with higher PhA values did (8388 mL ± 3168 mL vs 7417 mL ± 2459 mL, *P* = .0287). Additionally, upon admission to the intensive care unit postsurgery, patients with lower PhA scores had higher severity and organ failure index scores.^[27]^ Furthermore, higher PhA before cardiac surgery is correlated with greater LVEF.^[27]^ In another study involving hemodialysis patients, a lower PhA was predictive of intradialytic hypotension.^[28]^ These findings suggest that a higher PhA may indicate better hemodynamic stability during surgery, potentially protecting against AKI.

Second, PhA may be linked to markers of inflammation, which are known contributors to AKI following cardiac surgery.^[29]^ A retrospective cohort study revealed a moderate negative correlation between PhA and high-sensitivity C-reactive protein,^[30]^ which itself has been associated with AKI after coronary bypass surgery.^[31]^ PhA is also negatively correlated with tumor necrosis factor in patients with cardiovascular diseases.^[32]^ These findings suggest that a higher PhA might indicate a lower baseline inflammatory state and reduced perioperative inflammation induced by cardiac surgery, potentially lowering the risk of AKI.

With respect to other predictors of postoperative AKI, weight,^[33]^ and the GFR^[29]^ have been previously associated, which is consistent with the findings of our study. Notably, our study is the first to demonstrate the predictive value of the SPhA for AKI following cardiac surgery. SPhA could be integrated into clinical practice when studying interventions aimed at improving it and assessing a reduction in the incidence of AKI or other outcomes following elective cardiac surgery.

The strengths of our study include its prospective cohort design, which provides high-quality evidence on nonrandomizable variables. Our findings are applicable to the Latino population, which is conducted in a center serving 3 Mexican states. Additionally, PhA measurements by a single researcher via the BI minimized measurement bias. Most of these results align with the literature, with the novel addition of the SPhA as an AKI predictor.

Weaknesses include the single-center design, lower incidence of certain comorbidities such as COPD and LVEF < 35%, use of SCr-only criteria for AKI diagnosis potentially underestimating its incidence, and logistical challenges in patient recruitment.

## 5. Conclusion

A PhA ≤ 4.5° was not associated with an increased risk for AKI or mortality in the 7 days following cardiac surgery, but it did correlate with variables indicating fragility, such as older age, female sex, lower weight, and reduced grip strength. Conversely, a high SPhA (>−0.54), which indicates greater cellular integrity adjusted for age, sex, and BMI, may serve as a protective factor against AKI, particularly stage 1 AKI (KDIGO).

## Acknowledgments

We thank the members of the Department of Cardiac Surgery at the Specialties Hospital, National Western Medical Centre, Mexican Social Security Institute, for their support of patient recruitment.

## Author contributions

**Conceptualization:** Mauricio Carvallo-Venegas.

**Data curation:** Mauricio Carvallo-Venegas.

**Formal analysis:** Mauricio Carvallo-Venegas, Enrique Rojas-Campos.

**Funding acquisition:** Mauricio Carvallo-Venegas.

**Investigation:** Mauricio Carvallo-Venegas.

**Methodology:** Mauricio Carvallo-Venegas, Jorge Andrade-Sierra, Enrique Rojas-Campos.

**Project administration:** Jorge Andrade-Sierra, Adriana Banda-López.

**Resources:** Luis Alberto Evangelista-Carrillo.

**Software:** Enrique Rojas-Campos.

**Supervision:** Jorge Andrade-Sierra, Enrique Rojas-Campos, Rolando Claure-Del Granado, Miguel Medina-Pérez, Luis Gerardo González-Correa, José Ignacio Cerrillos-Gutiérrez, Adriana Banda-López, Ricardo Parra-Guerra, Alfredo Gutiérrez-Govea, Saúl Tejeda-del Toro, Laura Elizabeth Aguilar-Fletes, Francisco Gerardo Yanowsky-Escatell, Moises Cruz-Landino, Luis Alberto Evangelista-Carrillo.

**Validation:** Jorge Andrade-Sierra, Enrique Rojas-Campos, Rolando Claure-Del Granado, Miguel Medina-Pérez, Luis Gerardo González-Correa, José Ignacio Cerrillos-Gutiérrez, Adriana Banda-López, Ricardo Parra-Guerra, Alfredo Gutiérrez-Govea, Saúl Tejeda-del Toro, Laura Elizabeth Aguilar-Fletes, Francisco Gerardo Yanowsky-Escatell, Moises Cruz-Landino, Luis Alberto Evangelista-Carrillo.

**Visualization:** Rolando Claure-Del Granado, Adriana Banda-López.

**Writing – original draft:** Mauricio Carvallo-Venegas, Luis Gerardo González-Correa.

**Writing – review & editing:** Mauricio Carvallo-Venegas, Jorge Andrade-Sierra, Rolando Claure-Del Granado.

## Supplementary Material


